# Identification of collaborative cross mouse strains permissive to *Salmonella enterica* serovar Typhi infection

**DOI:** 10.1038/s41598-023-27400-1

**Published:** 2023-01-09

**Authors:** Kishore R. Alugupalli, Sudeep Kothari, Matthew P. Cravens, Justin A. Walker, Darren T. Dougharty, Gregory S. Dickinson, Louis A. Gatto, Andreas J. Bäumler, Tamding Wangdi, Darla R. Miller, Fernando Pardo-Manuel de Villena, Linda D. Siracusa

**Affiliations:** 1 Department of Microbiology and Immunology, Sidney Kimmel Cancer Center, Sidney Kimmel Medical College, Thomas Jefferson University, 233 South 10th Street, BLSB 726, Philadelphia, PA 19107 USA; 2 Vaccine Development Section, International Vaccine Institute, Gwanak-gu, Seoul, 151-919 Republic of Korea; 3 Department of Biological Sciences, SUNY at Cortland, Cortland, NY 13045 USA; 4 Department of Medical Microbiology and Immunology, School of Medicine, University of California, Davis, CA 95616 USA; 5 Department of Genetics and Lineberger Comprehensive Cancer Center, University of North Carolina, Chapel Hill, NC 27599 USA; 6 Department of Medical Sciences, Hackensack Meridian School of Medicine, Nutley, NJ 07110 USA

**Keywords:** Pathogenesis, Diseases, Infectious diseases, Immunology, Adaptive immunity, Vaccines, Microbiology, Pathogens

## Abstract

*Salmonella enterica* serovar Typhi is the causative agent of typhoid fever restricted to humans and does not replicate in commonly used inbred mice. Genetic variation in humans is far greater and more complex than that in a single inbred strain of mice. The Collaborative Cross (CC) is a large panel of recombinant inbred strains which has a wider range of genetic diversity than laboratory inbred mouse strains. We found that the CC003/Unc and CC053/Unc strains are permissive to intraperitoneal but not oral route of *S.* Typhi infection and show histopathological changes characteristic of human typhoid. These CC strains are immunocompetent, and immunization induces antigen-specific responses that can kill *S.* Typhi in vitro and control *S.* Typhi in vivo. Our results indicate that CC003/Unc and CC053/Unc strains can help identify the genetic basis for typhoid susceptibility, *S.* Typhi virulence mechanism(s) in vivo, and serve as a preclinical mammalian model system to identify effective vaccines and therapeutics strategies.

## Introduction

*Salmonella enterica* serovar Typhi (*S.* Typhi) is the causative agent of typhoid fever in humans. Global estimates reported by the CDC indicate that 21.6 million cases of typhoid fever occur each year resulting in 226,000 deaths^1^. The rapid emergence of multiple drug-resistant strains of *S.* Typhi now complicates the treatment of typhoid^2^. Typhoid is a vaccine-preventable disease and vaccination of high-risk populations, such as infants and young children, is considered the most promising strategy for control^3^. *S.* Typhi expresses Vi polysaccharide (ViPS), a well-known virulence factor^4,5^ and is a target for protective immune responses^3,6^. Three types of vaccines are currently available: (1) live attenuated vaccine, (2) subunit vaccines composed of plain ViPS^7,8^ and, (3) ViPS conjugated to a variety of carrier proteins such as rEPA, a recombinant Exoprotein A from *P. aeruginosa*^9^, CRM197, a non-toxic mutant of diphtheria toxin^10^, tetanus toxoid^11^, or diphtheria toxoid^12^.

Because *S.* Typhi is a human-restricted pathogen, vaccine efficacy data originates from clinical trials^9,13^. The live attenuated vaccine (Vivotif®) has an efficacy of ~ 60%^7,8^, does not express ViPS and is not recommended for children < 6 years of age due to safety concerns^7^. Subunit vaccines are safe for all ages, but the immune responses induced by plain ViPS vaccines, such as Typhim Vi® or Typherix®, are short-lived and their efficacy is ~ 55% in older children and adults^7^. Importantly, plain ViPS vaccines do not induce optimal antibody responses in children under 2 years of age. Fortunately, ViPS conjugated to rEPA, and tetanus toxoid can induce anti-ViPS responses in infants and young children with 80–90% efficacy^9,13^. Although antibodies are considered as the correlate of protection, the lack of a preclinical animal model makes it difficult to understand the variation in efficacy and the mechanism of protection conferred by these vaccines.

Ethical considerations make it difficult to fully characterize histopathology and immunology relevant to typhoid fever in humans^8,14^. A human *S.* Typhi challenge model was carried out in the UK, a non-disease endemic country. In this controlled challenge model, 4 of 40 participants fulfilled predetermined criteria for typhoid infection^15^. Nevertheless, this model enabled the accelerated testing of the newly WHO-prequalified ViPS-tetanus toxoid conjugate vaccine^16^. However, this human model will not provide an in-depth understanding of the factors involved in host susceptibility/resistance or the in vivo mechanism(s) of protective immunity to the extent that experimental animal models could provide. Commonly used inbred mouse strains e.g., C57BL/6J or BALB/cJ are not susceptible to *S.* Typhi infection. Previous research had demonstrated that *Tlr11*^*−/−*^ mice permit *S.* Typhi infection^17^; because humans do not have the gene-encoding for *Tlr11*, it was hypothesized that might account for the resistance of mice to *S.* Typhi. For reasons that are not clear, several other researchers including the original researchers, failed to reproduce *Tlr11*^*−/−*^ mouse permissiveness to *S.* Typhi^18,19^. Several research groups demonstrated that CD34^+^ human hematopoietic stem cell-engrafted NOD/SCID/IL-2Rγc^null^ or NOD/SCID/IL-2Rγc^null^ mice, referred to as “Human Immune System” or “humanized” mice are susceptible to *S.* Typhi infection^20–22^. Although humanized mice have the potential to understand typhoid pathogenesis, the humoral immunity of these mice is immature and such mice do not respond to ViPS immunization^23,24^. Therefore, the “humanized” mouse model in its current form is not appropriate for understanding immune responses to *S.* Typhi. Thus, there is a strong need to explore a fully immunocompetent and *S.* Typhi-permissive mouse model to decipher the mechanisms of typhoid vaccine-mediated protective immune responses.

Genetic variation in humans is far greater and more complex than that in commonly used inbred laboratory mice such as C57BL/6J, which may in part provide an explanation for differences in the infection susceptibility and progression in humans. The establishment of a murine model for human typhoid requires a system for the generation of mouse strains with great genetic diversity in which characterization can take place. A panel of recombinant inbred (RI) strains has been developed that incorporates a wider range of genetic diversity than inbred mouse strains. This set of RI strains is called the Collaborative Cross or CC^25^. The CC strains were derived from 8 genetically diverse inbred progenitor strains: A/J, C57BL/6J, 129S1/SvImJ, NOD/ShiLtJ, NZO/H1J, CAST/EiJ, PWK/PhJ and WSB/EiJ (https://csbio.unc.edu/CCstatus/index.py). The CC strains were designed specifically for complex trait analysis^25^. Each CC strain contains portions of the genomes of the 8 progenitor strains and each CC strain has been bred to homozygosity (> 90%). This new resource is supporting Systems Genetics and Systems Biology research at an unprecedented level. In fact, CC strains have been shown to serve as powerful model to identify host resistance and susceptibility alleles governing infections due to a variety of viral, bacterial and fungal pathogens^26^, including Influenza A virus^27^, SARS Corona virus^28^ Ebola virus^29^, *Mycobacterium tuberculosis*^30^, *Salmonella enterica* serovar Typhimurium (S. Typhimurium)^31,32^ and *Aspergillus fumigatus*^33^.

In the present study, we report the identification of immunocompetent *S*. Typhi-susceptible CC strains. The CC003/Unc and CC053/Unc strains are permissive to *S.* Typhi infection, intraperitoneally, but not through the oral route of infection. The CC mouse model described here will not only provide a much-needed preclinical tool for assessing the efficacy of vaccine candidates against typhoid but will also permit genetic analyses for *S.* Typhi susceptibility/resistance.

## Results

### CC mice are permissive to *S.* Typhi replication in vivo

**Figure 1:**
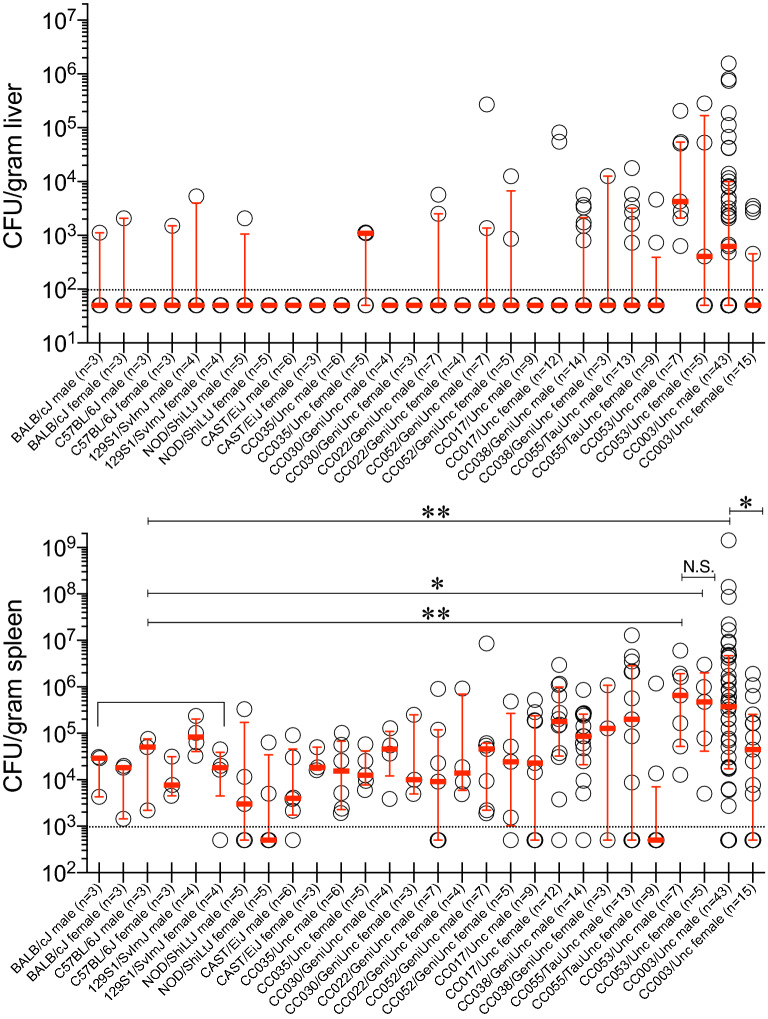
Bacterial burden in the liver and spleen of mice infected with *S.* Typhi. Inbred mouse strains, CC progenitors and CC strains of both sexes (age 8–12 weeks) were infected i.p. with 2 × 10^4^ CFUs of *S.* Typhi strain Ty2 in 100 μl of DPBS. Six days post-infection mice were sacrificed and bacterial burden in the liver and spleen was determined by plating serial tenfold dilutions of tissue homogenates followed by colony counting. Each open circle represents an individual mouse, the red bar represents median and interquartile range to identify statistical dispersion and range of susceptibility. Dotted line indicates the limit of detection. Statistics were performed using the Mann Whitney U test. ** = *p* < 0.01; * = *p* < 0.05; N.S. = not significant.

Since *S*. Typhi is a human-restricted pathogen, researchers often employ S. Typhimurium, a closely related bacterium that causes “typhoid-like” disease in mice. Organs of the mononuclear phagocytic system, such as spleen and liver, are the major sites of replication of *S.* Typhi in humans and this pattern is well-reflected in the i.p. route of infection of *S.* Typhimurium in mice^34,35^. To test whether CC mice are permissive or not, we infected 9 different CC mouse strains, 4 CC progenitors and BALB/cJ of both sexes i.p. with 2 × 10^4^ CFUs of *S.* Typhi strain Ty2. We assessed bacterial burden in the spleen 6 days post-infection to allow time for a detectable S. Typhi replication or host clearance. BALB/cJ, all 4 CC progenitor strains (C57BL/6J, 129S1/SvImJ, NOD/ShiLtJ, and CAST/EiJ), and several CC strains (CC035/Unc, CC030/GeniUnc, CC022/GeniUnc, and CC052/GeniUnc) showed little-to-no bacteria in the liver (Fig. 1). The bacterial count in the spleens of all these mice was lower than or close to the number of bacteria initially injected (Fig. 1). Some CC017/Unc females, CC038/GeniUnc and CC055/TauUnc mice showed a marginal increase in the bacterial burden in the liver and spleen (Fig. 1). Although an increase in bacterial load was not seen consistently in the livers of all CC003/Unc and CC053/Unc mice, the bacterial load in the spleens of these mice was significantly higher than that observed in the commonly used laboratory strains BALB/cJ, C57BL/6J, and 129S1/SvImJ (Fig. 1), indicating bacterial growth in these two CC strains. Interestingly, we observed a significant difference in the bacterial burden between male and female CC003/Unc mice (Fig. 1), a pattern not observed in the spleens of CC053/Unc mice (Fig. 1). These data suggest that CC053/Unc males and females, and CC003/Unc males but not females are permissive to *S.* Typhi replication in vivo.

### Histological features of *S.* Typhi infection in the livers of CC mice recapitulates that of human and murine typhoid pathology

**Figure 2:**
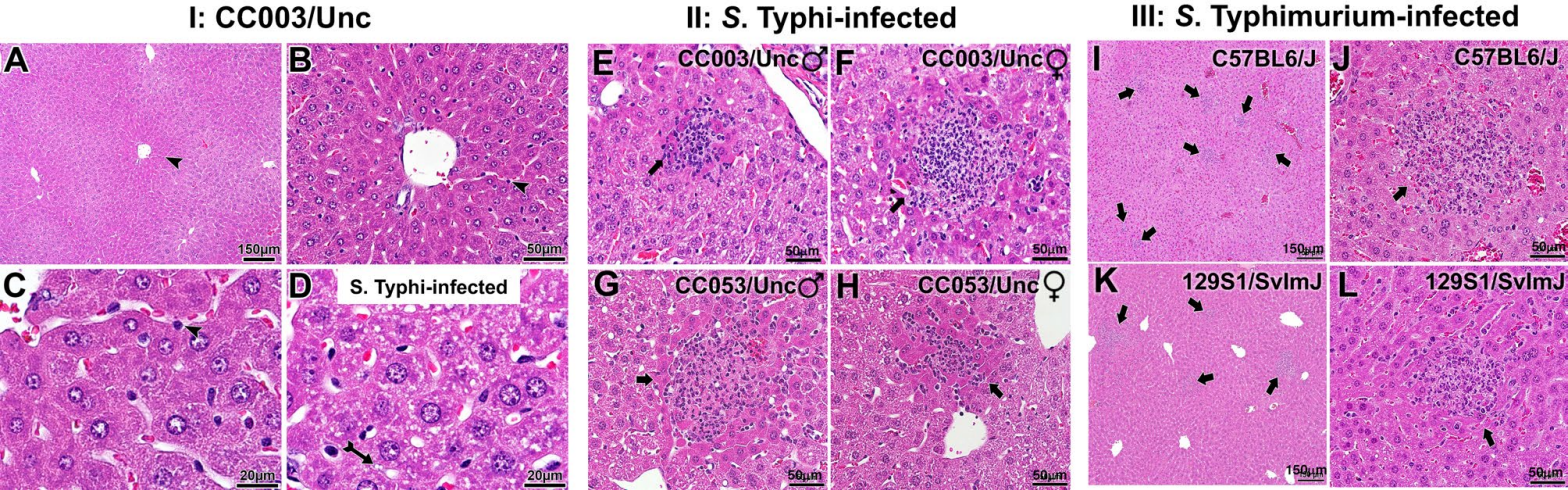
Histological features of *S.* Typhi infection in the livers of CC mice. Mice (age 8–12 weeks) were infected as in Fig. 1. The data presented is representative of five male and five female mice. Hematoxylin and eosin-stained liver sections of 6-day post-infected mice for *S.* Typhi and 3-day post-infected mice for *S.* Typhimurium were analyzed. Panel A, B and C show representative livers of the uninfected CC003/Unc mice at 3 different magnifications with an arrowhead pointing to a Kupffer cell. Panel D shows patterns of steatosis (indicated with an arrow) in infected mice, which is absent in uninfected mice (Panel C). Panels E–H depict representative liver sections of *S.* Typhi-infected male and female CC003/Unc and CC053/Unc mice. Panels I–L are liver sections of *S.* Typhimurium-infected C57BL/6J and 129S1/SvImJ mice at two different magnifications. Black arrows indicate lesions. Horizontal black bars in the right bottom corner show the size in μm.

Since preliminary screening of CC strains suggested the permissiveness to *S*. Typhi infection in CC003/Unc and CC053/Unc mice (Fig. 1), we examined hematoxylin and eosin-stained liver specimens of these two CC strains to characterize histopathology. We found that the livers of CC003/Unc mice had normal tissue architecture (Fig. 2A,B). However, upon *S.* Typhi infection the liver tissue showed signs of steatosis, an abnormal retention of lipids within hepatocytes (Fig. 2D), as compared to uninfected mice (Fig. 2C). This indicates a perturbation of liver metabolism in infected mice. Furthermore, *S.* Typhi-infected CC003/Unc and CC053/Unc mouse livers exhibited lesions that consist of mononuclear cell infiltrates, congestion of sinusoids, and altered staining with little/no steatosis in hepatocytes proximal to the lesion (Fig. 2E–H). While tissue biopsies are rare in typhoid-infected patients, biopsies have been collected in cases where typhoid is not initially considered and are used to aid diagnosis^36^. These rare cases provide some of the only information known about the histopathology of typhoid. Interestingly, the lesions observed in CC003/Unc and CC053/Unc mice (Fig. 2E–H) appear very similar to those described for human typhoid patients^37^. As a comparison, we also show that *S.* Typhimurium infection results in similar liver pathology that is commonly observed in inbred mice, C57BL/6J (Fig. 2I,J) and 129S1/SvImJ (Fig. 2K,L).

### CC003/Unc and CC053/Unc mice contain all the major subsets of cells of the innate and adaptive immune system

**Figure 3:**
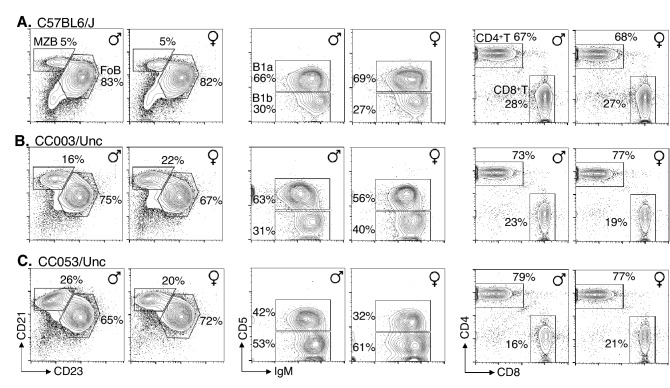
Flow cytometric analysis of mature lymphocyte subsets in CC mice. Spleen cells of mice (age 8–12 weeks) were stained with a cocktail of antibodies specific for CD19, B220, CD21, and CD23 or a cocktail of antibodies specific for CD3, CD4 and CD8, and analyzed by flow cytometry. Splenic B cells were first identified as CD19^+^ and B220^+^ cells (plots not shown) and were resolved further as MZ (CD23^lo^ and CD21^hi^) and Fo (CD23^hi^ and CD21^lo^) B cells, and their frequencies among CD19^+^ & B220^+^ cells are indicated within the plots. Splenic T cells were first identified as CD3^+^ (plots not shown) and resolved them into CD4^+^ and CD8^+^ cells. Since B1b and B1a cells are found in abundance in the coelomic cavity of mice, peritoneal cells were stained with antibodies specific for CD19, CD11b, surface IgM and CD5. All cells were first identified as B1 cells (CD19^+^ & CD11b^+^ cells [plots not shown]). The frequency of B1a (IgM^+^ & CD5^+^) and B1b (IgM^+^ & CD5^−^) subsets were shown. All data were generated by analyzing 50,000–100,000 cells and are representative of 3–5 mice from each mouse strain. Five percent contour plots are shown.

Susceptibility of mice to an infection can be due to immune deficiency. Since immune competency of CC mice is necessary for studying protective responses to *S.* Typhi in preclinical models, we performed a comprehensive flow cytometric analysis of cells in the spleen and coelomic cavity of CC003/Unc and CC053/Unc mice, as well as C57BL/6J mice as a control. In mice, the mature B cells can be divided into 4 subsets, namely, Fo, MZ, B1a, and B1b cells. Each of these subsets occupy a distinct functional niche in protective immunity^38^. For example, antibody responses to ViPS in mice are generated primarily by B1b cells^39^. We found that all 4 major B cell subsets including B1b are present in CC003/Unc and CC053/Unc mice and their frequency is within the range observed in C57BL/6J mice, except the frequency of MZ B cells, which is higher in the CC mice compared to C57BL/6J mice (Fig. 3A vs. B,C). The ratio of CD4 to CD8 T cells in C57BL/6J mice is typically 2–3:1^40^, and we found a slightly altered ratios in CC003/Unc and CC053/Unc mice (Fig. 3). It has been reported recently that there is a high variablility in the CD4 and CD8 ratios as well as total B cell frequencies in CC strains^41^. Furthermore, in the spleens of CC003/Unc and CC053/Unc mice, the frequency of natural killer (NK) cells (CD3^−^, NKp46^+^) neutrophils (CD11b^+^, Gr1^+^ & F4/80^−^), macrophages (CD11b^+^ & F4/80^+^), and dendritic cells (CD11b^+/lo^, CD11c^+^ & F4/80^−^) were 2–4%, 1.5–2.5%, 4–9%, and 5–9%, respectively. These frequencies are within the range reported previously for C57BL/6, BALB/c and 129/Sv mice^40^. These data indicate that all the major innate and adaptive immune cell compartments are intact in CC003/Unc and CC053/Unc mice, and therefore enabling the study of protective immune responses against *S.* Typhi in vitro and in vivo.

### CC003/Unc mice are immunocompetent

**Figure 4:**
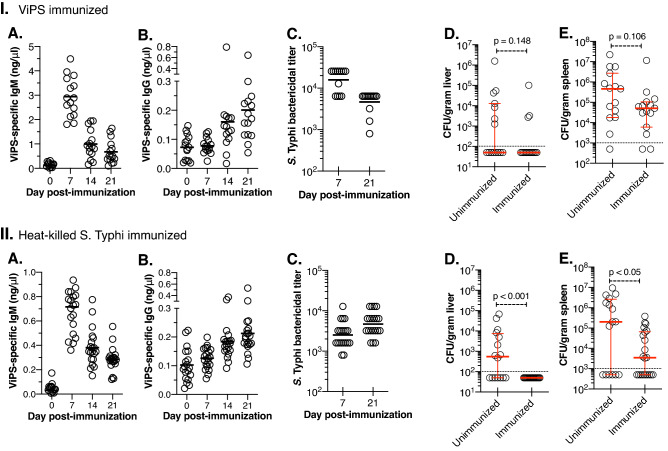
CC mice are immunocompetent. Eight to twelve-week-old CC003/Unc male mice were immunized i.p. either with (**I**) 2.5 μg ViPS or (**II**) 3 × 10^8^ CFUs of heat-killed *S.* Typhi strain Ty2 in 100 μl of DPBS. ViPS-specific IgM (A) and IgG (B) on indicated time points were measured by ELISA. Each open circle represents an individual mouse. The solid bar represents mean values. (C) Serum bactericidal antibody titers against live *S.* Typhi strain Ty2 were determined at 7- and 21-days post-immunization. (D,E) Unimmunized and immunized mice (21 days post-immunization) were infected i.p. with 2 × 10^4^ CFUs of *S.* Typhi strain Ty2 in 100 μl of DPBS. Six days after challenge, bacterial burden in liver and spleen were determined as in Fig. 1. The dotted line in panels D&E indicates detection limits. Data for all figures is a pool of minimum of two independent experiments. Statistical differences were determined by the Mann–Whitney U test.

Among the 9 CC strains of both sexes screened, CC003/Unc male mice showed relatively high susceptibility to *S.* Typhi replication as determined by the bacterial load in the liver and spleen in more than 6 independent experiments comprising of a total of 43 mice (Fig. 1). To test the proof of principle that CC mice can generate protective immunity against *S.* Typhi, we chose the CC003/Unc strain to test vaccination efficacy. We immunized male CC003/Unc mice with either plain ViPS or heat-killed *S.* Typhi. We found that both types of immunizations induced a robust anti-ViPS IgM response that peaked at 7 days post-infection (Fig. 4IA,IIA) as in immunocompetent C57BL/6J mice^35^ and the serum obtained from the CC003/Unc mice at this time point was capable of killing *S.* Typhi in vitro in a complement-dependent serum bactericidal assay (Fig. 4IC,IIC). IgM response typically declines after 7 days post-infection, which is concurrent with the induction of IgG response due to antibody isotype switching^35^. Both immunizations generated an anti-ViPS IgG response by 21 days post-infection (Fig. 4IB,IIB), and serum obtained at this time point also killed *S.* Typhi in vitro (Fig. 4IC,IIC). These data suggest that CC003/Unc mice undergo isotype switching normally and the antibodies generated by these immunizations can provide protection. To test whether anti-ViPS antibodies confer protection in vivo, we challenged male CC003/Unc mice with *S.* Typhi strain Ty2 and measured bacterial load 6 days later as in Fig. 1. Although ViPS-immunized mice exhibited a reduced bacterial burden in the liver and spleen compared to unimmunized mice, the difference was not statistically significant (Fig. 4ID,E), consistent with the low efficacy of the plain ViPS vaccine^7^. Immunization with whole bacteria induces a qualitatively different antibody response^42^. In a *S.* Typhimurium “surrogate” challenge model, we have previously shown that immunization with heat-killed *S.* Typhi controls bacterial burden better than ViPS immunization^35^. Here we found that immunization of CC003/Unc mice with heat-killed *S.* Typhi confers more efficient protection compared to ViPS, as determined by a significant decrease in bacterial burden in the liver and spleen (Fig. 4IID,E).

**Figure 5:**
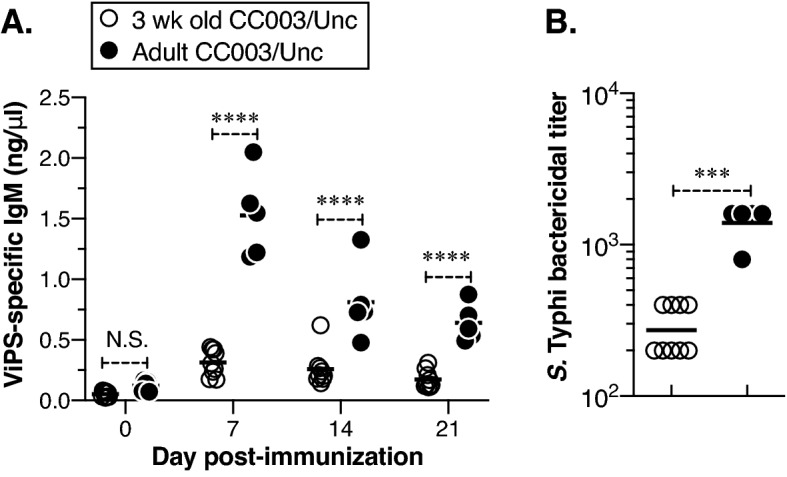
Young CC003/Unc mice do not respond as efficiently as adult CC003/Unc to ViPS immunization. (**A**) Three-week-old (young) or 8- to 12-week-old (adult) CC003/Unc mice were immunized with ViPS as in Fig. 4, and the levels of ViPS-specific IgM and IgG were measured by ELISA. Statistical differences were determined using two-way ANOVA. Each open circle represents a young mouse, and each filled circle represents an adult mouse. The bar represents the mean. (**B**) Serum bactericidal titers against *S.* Typhi strain Ty2 were determined at 7 days post-immunization. Statistical differences were determined by the Mann–Whitney U test. The bar represents the geometric mean. The data are pooled from two independent experiments. ***p < 0.001, ****p < 0.0001. N.S., statistically not significant.

Unlike adults, young children and infants do not respond to polysaccharide antigens such as ViPS. We have previously shown that 3-week-old mice (like young children) do not respond to ViPS efficiently due to a restricted antibody repertoire^43^. Therefore, we compared antibody responses to ViPS in young (3-week-old) and adult (8–12-week-old) CC003/Unc mice. We found that compared to adult CC003/Unc mice, the 3-week-old CC003/Unc mice are not capable of mounting an efficient IgM response to ViPS (Fig. 5A) and their serum was inefficient in killing *S.* Typhi in vitro (Fig. 5B). In summary, this data demonstrate that CC mice are not only immunocompetent but can control *S.* Typhi burden upon immunization and suggest that both young and adult CC003/Unc mice can serve as an experimental system to identify novel typhoid vaccines and therapeutics.

## Discussion

One of the barriers to advancing the treatment and prevention of typhoid is the lack of a suitable animal model to study *S.* Typhi infection^8^. Since *S*. Typhi is strictly human adapted, *S.* Typhimurium, a natural pathogen of mice became the most widely used bacterium to understand pathogenesis and immunity in several inbred strains (e.g., C57BL6/J and 129/Sv). *S.* Typhimurium causes a “Typhoid-like” systemic disease in mice and shares 90% of genes with *S.* Typhi^44^. However, ~ 600 *S.* Typhi genes, including those encoding for ViPS biogenesis and typhoid toxin, are not found in *S.* Typhimurium^44^. Furthermore, several genes found in *S.* Typhimurium are pseudogenes in *S.* Typhi^45^. Therefore, wildtype *S.* Typhimurium strains cannot serve as a synonymous model system to decipher the role of *S.* Typhi-specific virulence factors. To understand the function of ViPS in vivo*,* Baumler and coworkers introduced all the *S.* Typhi genes required for ViPS biogenesis in *S.* Typhimurium^46^. Using this “chimeric” *S.* Typhimurium strain e.g. TH170^46^, a role for ViPS in resisting C3 deposition and complement receptor 3-mediated clearance^47^, in evading of TLR4 recognition^48,49^, and microbe-guided neutrophil chemotaxis was identified^50,51^. The length of LPS of *S.* Typhimurium is very long compared to that of *S.* Typhi. In *S.* Typhimurium the length of LPS is controlled by the *FepE* gene product, which is a pseudogene in *S.* Typhi. To mimic the surface characteristics of *S.* Typhimurium to resemble that of *S.* Typhi, the *S.* Typhimurium strain TH170 was further engineered by deleting the *FepE* gene^4^. This strain of *S.* Typhimurium, referred to as RC60, was shown to exhibit cell surface and other characteristics of *S.* Typhi^4^. Using the *S.* Typhimurium strain RC60, we were able to identify several aspects of the anti-ViPS antibody repertoire required for protective immunity in vivo^35,43,52^. Thus, the use of *S.* Typhimurium as a “surrogate” model to understand certain virulence mechanisms of *S.* Typhi has been justified. However, the heterologous expression of *S.* Typhi genes in *S.* Typhimurium may not always permit identification of the role of certain *S.* Typhi-specific components. For example, the typhoid toxin of *S.* Typhi appears to be host-adapted and binds to N-acetylneuraminic acid (Neu5Ac) that is abundantly expressed in humans but not in mice^53,54^. Therefore, studying the role of typhoid toxin in the pathogenesis requires a permissive mouse model that contains human tissue, such as “humanized” mice. Interestingly, a genome-wide analysis of *S.* Typhi mutants in “humanized" mice confirmed that ViPS is essential for virulence but surprisingly not the typhoid toxin^55^. The controlled human infection model also revealed that typhoid toxin is neither required for infection nor for the development of early typhoid fever symptoms^56^. Since the human challenge model represents early events of typhoid, the role of typhoid toxin in severe disease or the establishment of bacterial carriage remains to be determined.

Host genetics clearly play a role in a variety of infectious diseases, as evidenced by hypothesis-driven analyses of polymorphisms in the *Tlr4* gene^57^. In fact, CC mice were developed as a resource for mammalian systems genetics^25^. Few susceptibility genes e.g., *Slc11a1* and *Tlr11* have been implicated for the permissiveness of *S.* Typhi. Notably, the relative susceptibility or resistance to *S.* Typhimurium, a close relative of *S.* Typhi, was shown to depend upon a single nucleotide polymorphism in the *Slc11a1* gene that encodes an ion transporter commonly referred to as Natural resistance-associated macrophage protein 1 (Nramp1)^58^ and neutrophil cytosolic factor 2 (Ncf2)^59^. In the UNC systems genetics database, (https://csbio.unc.edu/CCstatus/index.py), we found that both CC003/Unc and CC053/Unc mice have the *Slc11a1* resistant allele inherited from the progenitor A/J and the ncf2 allele is not mutated^60^. Although the role of *Tlr11* is an ongoing controversy^18,19^, we have not found any mutation in the *Tlr11* gene either in CC003/Unc or CC053/Unc mice. Therefore, a comprehensive genetic approach may help us discover novel genes responsible for susceptibility to *S.* Typhi in CC003/Unc and CC053/Unc. For example, one approach is to perform classical genetic crosses to generate F1 hybrids and F2 offspring by crossing either CC053/Unc or CC003/Unc with BALB/cJ, a known *S.* Typhi resistant and a non-CC progenitor strain, respectively. The susceptibility of F1 hybrids and F2 offspring to *S.* Typhi can be tested as in Fig. 1, and the genotype of those mice can be determined using the Giga Mouse Universal Genotyping Array (GigaMUGA)^61^ or MiniMUGA^62^ to find associations between resistance/susceptibility phenotype and genotype. Indeed, using a combination of genotyping and quantitative trait loci mapping, several *S.* Typhimurium infection susceptibility loci have recently been identified in CC042/GeniUnc mice^32^. Although CC042/GeniUnc mice inherited the susceptible *Slc11a1* locus from the C57BL/6 progenitor and its *Tlr4* gene product is functional, the bacterial burden in the CC042/GeniUnc mice was 1000-fold more than that in C57BL/6N mice, suggesting that the hyper-susceptibility is due to other factors. Interestingly, CC042/GeniUnc mice also showed lower spleen weights and decreased B, T, and myeloid cell populations compared to control C57BL/6N mice, suggesting that an abnormality in the architecture of the immune system or a partial immune deficiency may be responsible for the hyper-susceptible phenotype. Indeed, an F2 cross between CC042/GeniUnc and C57BL/6N mice identified a susceptibility locus accompanied by a loss-of-function variant of the integrin alpha L (*Itgal*) gene^31^, which encodes for LFA-1, a molecule central to immune cell adhesion and trafficking. This hyper-susceptibility phenotype has been confirmed independently by comparing *S.* Typhimurium infection in C57BL/6N and LFA-1^−/−^ mice^31^. Unlike the CC042/GeniUnc mouse system for *S.* Typhimurium hyper-susceptibility, the reason for CC003/Unc and CC053/Unc mice susceptibility is unlikely to be associated with an altered architecture/composition of the immune system. The *Itgal* gene of CC003/Unc and CC053/Unc mice is inherited from C57BL/6J and 129S1/SvImJ progenitors, but not from WSB/EiJ progenitor as in CC042/GeniUnc mice. Most importantly, CC003/Unc and CC053/Unc mice are immunocompetent (unlike CC042/GeniUnc or “humanized” mice) and possess all the major populations of innate and adaptive immune cells (Fig. 3) and respond to both T cell-independent immunogen (i.e., isolated plain ViPS) or T cell-dependent immunogen (i.e., heat-killed *S.* Typhi) (Fig. 4).

Feaco-oral route is a natural mode of *S.* Typhi transmission in humans, that eventually results in a systemic infection. When we attempted oral infection using a gavage needle (10^9^ CFU of strain Ty2 in 100 μl PBS), we did not observe permissiveness of *S.* Typhi infection in CC003/Unc mice. Therefore, we chose i.p. infection which is commonly used method for sytemic infection in a variety of infectious disease models in mice. This suggests that the CC003/Unc mice do not capture certain aspects of typhoid disease that occurs in humans, such as intestinal pathology, and bacterial shedding in faeces. Screening more CC strains to *S.* Typhi susceptibility might help identify specific CC strains that can capture the oral infection characteristics and intestinal pathology seen in humans.

In conclusion, the immunocompetent, *S.* Typhi permissive CC mouse model using CC003/Unc and CC053/Unc presented here can provide an in vivo experimental system to evaluate novel preventive and therapeutic interventions. Additionally, genomic analyses of the CC mouse model can help us understand why responses to different ViPS conjugate vaccines vary among populations in disease-endemic countries^10^ and why some individuals become chronic and asymptomatic carriers for spreading typhoid. This CC mouse model system can also enable the *S.* Typhi research community to identify putative *S.* Typhi virulence factor(s) and characterize their role in the progression of typhoid.

## Methods

### Mice

The Thomas Jefferson University (TJU) Institutional Animal Care and Use Committee have approved these studies. All methods were performed in accordance with the guidelines and regulations of TJU Institutional Biosafety Committee. Reporting of the animal experiments followed the recommendations in the ARRIVE guidelines. C57BL/6J, 129S1/SvImJ, BALB/cJ, NOD/ShiLtJ and CAST/EiJ were purchased from The Jackson Laboratory (Bar Harbor, ME). All CC strains used were purchased in 2014–2015 from the Systems Genetics Core Facility, University of North Carolina at Chapel Hill (Chapel Hill, NC). Mice were housed in micro-isolator cages with free access to food and water and were maintained and bred in a specific pathogen-free facility at TJU. Three-week-old mice were considered young and 8–12-week-old mice were considered adult.

### Infection

For mouse infections *S.* Typhi strain Ty2, a well-studied strain (obtained from Dr. Andreas Baumler) was grown to an OD_600_ of ~ 1.0 in Luria Bertani (LB) broth containing 10 mM NaCl. Bacteria were washed twice in Dulbecco’s phosphate-buffered saline (DPBS); and bacterial density was adjusted to 2 × 10^5^ colony forming units (CFU)/ml. Mice were infected intraperitoneally (i.p.) with 2 × 10^4^ CFU in 100 μl of DPBS. Because organs of the mononuclear phagocytic system, such as spleen and liver, are the major sites of replication of *S.* Typhi in humans, we assessed bacterial burden in these organs 6 days later to allow time for a detectable *S.* Typhi replication or host clearance. On day 6 post-infection, liver and spleen were collected and tissues were processed using a Minilys tissue homogenizer (Bertin Technologies, Montigny-le-Bretonneux, France). Bacterial burden in these tissue homogenates was measured by counting CFU on LB agar plates.

### Histopathology analysis

Liver tissues obtained on day 6 post-infection were fixed in 10% buffered formalin and 4 μM paraffin-embedded sections were stained with hematoxylin and eosin. The specimen slides were scanned at 20 × magnification on Aperio CS2 Scanscope® (Leica Biosystems Inc.) followed by observer-blind histopathological analysis.

### Flow cytometry

Peritoneal cavity cells were harvested to determine the frequency of B1a and B1b cells, and spleen cells were harvested to determine the frequency of follicular (Fo) B, marginal zone (MZ) B, CD4^+^T, CD8^+^T, Natural Killer (NK), dendritic cells, neutrophils, and macrophages from individual mice. The cell concentrations were adjusted to 2.5 × 10^7^ cells/ml in staining medium [Minimum Essential Medium Eagle with Earle’s salts and without L-glutamine or phenol red (Corning Cellgro, Manassas, VA) with 3% newborn calf serum (HyClone Laboratories Inc. Logan, UT), 1 mM EDTA]. Cell suspensions were incubated with 2.4G2 antibody for 15 min to block Fc receptors and stained with appropriate antibody cocktail. The antibodies, anti-CD11b-AF700 (clone: M1/70), anti-CD5 Pacific Blue (clone: 53–7.3), anti-CD19-PE-Cy7 (clone: eBio1D3) were purchased from eBioscience (San Diego, CA); anti-B220-PercP-Cy5.5 (clone: RA3-6B2) was purchased from Caltag (Burlingame, CA); anti-IgM-PE (clone: 1B4B1) and CD23-PE (clone: B3B4) was purchased from BD Pharmingen (San Jose, California). Anti-CD3 FITC (clone:17A2), anti-CD4-BV605 (clone: RM4-5), anti-CD8-APC-Cy7 (clone: 53–6.7), anti-NKp46-APC (clone: 29A14), anti-F4/80-FITC (clone: BM8E), anti-GR1-Pacific blue (clone: RB6-8C5), anti-CD11c-PE-Cy7 (clone: N418) and anti-CD21/35-APC (clone: 7E9) were purchased from Biolegend. (San Diego, CA). After staining, cells were washed twice with staining medium and the preparations were analyzed on an LSRII™ flow cytometer (Becton Dickinson, Mountain View, CA) using the FACS Diva™ software (Becton Dickinson). Data were analyzed using the FlowJo™ software (Treestar, San Carlos, CA).

### Immunization

Two and a half μg of Vi Polysaccharide (ViPS; Lot 5 PDMI 158,299 obtained from the U.S. Food and Drug Administration, Silver Spring, MD) dissolved in 100 µl DPBS was used to immunize mice i.p. For whole bacterial immunization, mice were injected i.p. with 3 × 10^8^ CFUs of heat-killed *S.* Typhi strain Ty2 in 100 µl DPBS^35^. The expression of ViPS is confirmed by serologically by slide agglutination test using a commercial Vi monoclonal antibody reagent (Statens Serum Institut diagnostica A/S, Denmark; Lot 188L-8). We also perform serum bactericidal assay using anti-*S*. Typhi human IgG (Lot R1, 2011; U.S. Food and Drug Administration, Silver Spring, MD) as described^35,52,63^. Blood samples were obtained 0-, 7-, 14- or 21-days following immunization and stored at − 20 °C.

### Enzyme-linked immunosorbent assay (ELISA)

ViPS-specific IgM and IgG were measured by coating 96-well microtiter plates (Nunc MaxiSorp™; Invitrogen, Carlsbad, CA) with 2 µg/ml of ViPS purified from *S.* Typhi clinical isolate C6524^64^ in DPBS overnight at room temperature. All plates were washed and blocked with 2% Bovine serum albumin (BSA) in PBS pH 7.2 (blocking buffer) for 2 h at room temperature. Blood from immunized mice was diluted to 1:25 for IgG detection and 1:50 for IgM detection in blocking buffer; ViPS-specific mouse IgM and IgG levels were interpreted as ng/μl “equivalents” using normal mouse serum standards (Bethyl Laboratories, Montgomery, TX), as described previously^35^.

### Serum bactericidal assay (SBA)

SBA was performed as previously described^35^. In brief, log-phase cultures (OD_600_ of 0.5 at 37 °C) of *S.* Typhi strain Ty2 were prepared in LB broth with 10 mM NaCl. Bacterial cells were washed in DPBS, and the bacterial cell density was adjusted to 1–3.5 × 10^4^ CFU/ml in DPBS. Serum samples were heat-inactivated by incubating at 56 °C for 30 min prior to use in the assay. Ten microliters of *S*. Typhi cells in DPBS (100–350 CFU) were added to each well of a round-bottom polypropylene 96-well plate containing 50 μl of heat-inactivated serum in serial dilutions, 12.5 μl baby rabbit complement (Pel-Freeze, Rogers, AR), and 27.5 μl DPBS. Triplicate samples of each dilution were incubated for 120 min at 37 °C with gentle rocking, and 10 μl of this mixture was plated on LB agar plates for enumerating bacterial CFU. Serum bactericidal antibody titers were defined as the reciprocal of the highest dilution that produced > 50% killing in relation to control wells containing complement, but no mouse serum.

### Statistical analysis

Data presented throughout depict pooled data from at least two independent experiments unless otherwise noted. Statistics were performed using the Prism 5 software program (GraphPad Software, Inc., La Jolla, CA).

## Data Availability

All data generated or analysed during this study are included in this published article.
